# Supplementing transcranial direct current stimulation to local infiltration series for refractory neuropathic craniocephalic pain: A randomized controlled pilot trial

**DOI:** 10.3389/fneur.2023.1069434

**Published:** 2023-03-01

**Authors:** Jan D. Wandrey, Joanna Kastelik, Thomas Fritzsche, Claudia Denke, Michael Schäfer, Sascha Tafelski

**Affiliations:** Department of Anesthesiology and Intensive Care Medicine, Charité—Universitätsmedizin Berlin, Corporate Member of Freie Universität Berlin and Humboldt-Universität zu Berlin, Berlin, Germany

**Keywords:** tDCS, chronic pain, neuropathic pain, craniocephalic pain, infiltration series

## Abstract

**Background:**

Some patients with neuralgia of cranial nerves with otherwise therapy-refractory pain respond to invasive therapy with local anesthetics. Unfortunately, pain regularly relapses despite multimodal pain management. Transcranial direct current stimulation (tDCS) may prolong pain response due to neuro-modulatory effects.

**Methods:**

This controlled clinical pilot trial randomized patients to receive anodal, cathodal or sham-tDCS stimulation prior to local anesthetic infiltration. Pain attenuation, quality-of-life and side effects were assessed and compared with historic controls to estimate effects of tDCS stimulation setting.

**Results:**

Altogether, 17 patients were randomized into three groups with different stimulation protocols. Relative reduction of pain intensity in per protocol treated patients were median 73%, 50% and 69% in anodal, cathodal and sham group, respectively (*p* = 0.726). Compared with a historic control group, a lower rate of responders with 50% reduction of pain intensity indicates probable placebo effects (OR 3.41 stimulation vs. non-stimulation setting, NNT 3.63). 76.9% (*n* = 10) of tDCS patients reported mild side-effects. Of all initially included 17 patients, 23.5% (*n* = 4) withdrew their study participation with highest proportion in the cathodal group (*n* = 3). A sample size calculation for a confirmatory trial revealed 120 patients using conservative estimations.

**Discussion:**

This pilot trial does not support series of anodal tDCS as neuro-modulatory treatment to enhance pain alleviation of local anesthetic infiltration series. Notably, results may indicate placebo effects of tDCS settings. Feasibility of studies in this population was limited due to relevant drop-out rates. Anodal tDCS warrants further confirmation as neuro-modulatory pain treatment option.

## 1. Introduction

Pain in the head-neck area can be debilitating for patients (1). Chronic neuropathic pain in particular can be a relevant factor contributing to global burden of disease (2, 3). First line treatment for patients with neuropathic pain includes medications such as gabapentinoids, duloxetine and tricyclic antidepressants together with adjunctive therapies such as physical and psychological therapy (4–6). Management of these patients can be challenging, and sometimes pain remains refractory to non-invasive treatment (7). In selected patients, interventional procedures provide alternative treatment options (4, 8). Although under debate, infiltration series with ganglionic and nerve blocks are commonly used by pain physicians (9). Results from our previous retrospective study indicate relevant beneficial effects of these infiltration series in multimodal therapy concepts: in a cohort of 83 patients with chronic neuropathic pain in the head-neck area refractory to standard treatment, a reduction of pain intensity on the numeric rating scale (NRS, 0-10) was achieved by mean 3.2 points (SD 3.3, *p* < 0.001) (10). Furthermore, about half of the included patients achieved clinically relevant improvement in NRS scores with a reduction of pain intensity by 30–50%. In this study, we used infiltrations at sphenopalatine ganglion (SPG), superior cervical ganglion and stellate ganglion, peripheral nerve blocks at occipital nerve and trigeminal nerve as described in the literature (10–12).

Recent fMRI studies support the use of infiltration series, since they change resting state functional activity in domains relevant for pain. Single infiltrations seem to facilitate small network changes (13) which may relate to relatively small clinical effects provided by single interventions (14). For longer lasting effects, repetitions of infiltrations were advocated (13, 15). In contrast, there are only few reported long-term effects on pain (10, 16). This could be due to maladaptive structural plasticity and neuronal reorganization (17). Hence, central modulation of this reorganization in addition to the peripheral infiltration series, is required.

Non-invasive brain stimulation is an emerging field in clinical research (18, 19). Though exact neurobiological mechanisms are still unclear, results from *in vitro* and *in vivo* studies suggest long term central changes of repetitive transcranial direct current stimulation (tDCS) mediated by metaplasticity rather than long-term potentiation or depression as discussed earlier in the literature (20). This metaplasticity includes changes in cellular mechanisms [e.g., effects of tDCS on excitatory synaptic efficacy (21)], neurotransmission [e.g., motor cortex excitability (22)] and effects on the neuroinflammatory system [e.g., anodal tDCS induced stimulation of neural stem cell migration and cathodal tDCS induced stimulation of neuroinflammatory response (23)] (20). The use of tDCS may be an option for pain disorders (18), oro-facial pain disorders in particular (24). Treatment with tDCS reorganizes neuronal representation of pain (25) and modulates maladaptive plasticity (18, 26). A recent study suggests changes in maladaptive corticomotor excitability by tDCS and thus leading to anti-nociceptive effects (27). A different approach discusses that the effects on pain by non-invasive brain stimulation are mediated by top-down modulation and restoration of defective endogenous inhibitory pain pathways (28).

tDCS has been used in studies in a variety of conditions focussing on pain, neurological and psychiatric diseases (29). A higher evidence level of recommendation in favor of treatment with tDCS was found for depression, craving and fibromyalgia (29).

Depending on polarity, tDCS either enhances (anodal tDCS) or reduces (cathodal tDCS) motor cortical excitability measured in motor-evoked potentials (MEP) (22, 30). Though anodal stimulation seems more promising, evidence suggests efficacy in pain treatment for both anodal (31, 32) and cathodal (33) tDCS above the M1 area.

Not only the direction of the current but also the electrode montage is critical in tDCS (34). In pain processing, a large network of different pain processing sites is activated and called the pain neuromatrix (32, 33, 35, 36). The superficial parts of the pain neuromatrix include the primary sensory cortex (S1), primary motor cortex (M1), and dorsolateral prefrontal cortex (DLPFC), making them the most common montage settings of tDCS in the literature (30). Compared to anodal tDCS of S1 and DLPFC, M1 stimulation seems to be the best spot to enhance brain excitability (32). Thus, anodal M1 stimulation is the most used and most promising stimulation side (30).

Since it appears to be safe, the use of tDCS additional to other treatment is common (37). For instance, a recent meta-analysis showed moderate to large effects of combined intervention of exercise with anodal tDCS on motor cortex compared to sham and exercise in chronic pain (38). Both, infiltration techniques (13) and tDCS (25, 39) seem to change resting state functional connectivity. Synergistic interactions in neuronal networks are a potential target by tDCS and infiltration series (18). To date, there is no data on a combination of these two interventions on pain intensity.

Therefore, this pilot study was performed to investigate trial feasibility, individual course of pain and pain relief, associated symptoms and side effects of tDCS and subsequent local infiltration series in patients with refractory cranial neuropathic pain syndromes.

## 2. Materials and methods

This prospective study was conducted in chronic pain patients at the pain outpatient center of the Charité-Universitätsmedizin Berlin, Campus Virchow Klinikum. The department provides clinical care for chronic pain patients and is run by a team of pain specialists, behavioral psychologists and trained pain nurses. All patients treated in the department from June 2016 to March 2019 were screened for eligibility. For inclusion, a clinical diagnosis of a condition according to the ICHD3 [chapter *13. Painful lesions of the cranial nerves and other facial pain*, e.g., trigeminal-neuralgia, post-herpetic trigeminal neuralgia, persistent idiopathic facial pain (PIFP)] was mandatory (40). Furthermore, only patients receiving local infiltration series for treatment of these cranial conditions were included. Infiltration series were performed based on judgement of attending physician and following standardized infiltration protocol (11, 41). Infiltration techniques used in this study are reported in detail in a previous study (10).

Exclusion criteria were patients under the age of 18, current reported pregnancy, accommodation in an institution due to an official or judicial order, patients participating in another trial during this study and patients with contraindications for tDCS (e.g., epilepsy, metal implants in stimulation area and implanted defibrillators). Patients fulfilling inclusion criteria were offered to participate in the study to receive additional tDCS application before each local infiltration. Eligible patients were asked for written informed consent. All infiltration series were performed as part of a multi-modal therapy concept following current recommendations (42, 43). This study uses some of the methods of our previous study and thus the methods description partly reproduces their wording (10). This study was approved by the Charité ethics committee (EA1/031/16) and followed the rule of the declaration of Helsinki in its updated 2013 version (44). Moreover, the study was registered (ClinicalTrials: NCT02747758) and applied the CONSORT checklist (45).

### 2.1. Study design

Patients were planned to receive 10 consecutive sessions of the infiltration and stimulation series with 48–72 h between each session following recommendations for long-lasting after-effects (30, 46–48). Before each session, the attending physician decided in a context-sensitive approach if a continuation of infiltration and thus stimulation series was indicated. If applicable, first tDCS (anodal/cathodal/sham, depending on study group) and afterwards local infiltration was performed. After completion of series, patients were followed-up for 6 months.

### 2.2. Outcome parameter

The primary outcome parameter was relative pain intensity reduction after completion of therapy series (typically after 2 weeks of treatment) measured in NRS score as a numeric value between 0 and 10. The secondary outcome parameter include absolute and relative pain reduction measured in NRS score after completion of tDCS stimulation vs. initial NRS score measured before stimulation and time until patients need additional regional-anaesthesiological interventions. The number of required regional-anaesthesiological interventions to achieve sufficient pain reduction and the analysis of adverse reaction (skin redness, headache, concentration, other) were other secondary outcome parameter.

We further evaluated patients' conditions, the used blockade technique, the response rate and the effect of sole tDCS stimulation. In addition to that, analyses of side-effects, drop-outs, self-assessment and a *post-hoc* sample size calculation was conducted.

### 2.3. Assessment of pain

For pain assessment, two assessment tools were used: the pain assessment protocol for infiltration series and the German Pain Questionnaire [daily report form, version 2007 (49)]. The assessment protocol was the basis of the evaluation of the primary outcome parameter and contained the NRS on an 11-point Likert scale (0–10) for static (at rest) and dynamic (maximum pain in stress) pain before and after tDCS. The German Pain Questionnaire was used both during stimulation and infiltration series and for the Follow-Up. Its core questions are derived from the grading of chronic pain status (50). They consist of four questions on a 11-point Likert scale (0–10): average pain in the last week, maximum pain in the last week, mental distress and impairment in daily activities. Furthermore, the German Pain Questionnaire consists of a question regarding the endurance of pain (1 = not applicable, I have no pain, 2 = I can tolerate it well, 3 = I can just tolerate it, 4 = I can tolerate it badly). For the pain assessment, patients were asked to name the most predominant painful side.

### 2.4. tDCS

Before each local anesthetic infiltration series, a 20 min tDCS stimulation was performed using the NeuroConn DC Stimulator^®^ with saline soaked, square sponge electrodes (surface 25cm^2^) similar to Nitsche and Paulus et al. (22, 47, 48) (see Supplementary Figure 1). Following the protocol of Morosoli et al., we placed the anode electrode over the primary motor cortex (M1) contralateral to the most predominant painful side, and the cathode electrode over the contralateral supraorbital area (51). The primary motor cortex is located in the Brodman location 4 (52, 53). Electrode position of C3,4 correlates with Brodman location 4, which is located in the precentral gyms, shoulder to wrist area, caudal to middle frontal gyrus (54). We determined the C3 or C4 placement using the recommendation of Jasper (55).

The patients were divided in three subgroups with either anodal, cathodal or sham stimulation. The same stimulation setting was used in every subgroup. Due to the triple-blinding study design, electrode placement was identical in either anodal or cathodal stimulation group with inverse current flow, depending on study allocation (e.g., anodal stimulation: anode=anode and cathode=cathode whereas cathodal stimulation anode=cathode and cathode=anode).

In the active groups (anodal and cathodal), stimulation started with an initialization phase with increasing current over 30 s. Afterwards, tDCS with 2 mA was applied for 20 min, following a phase with decreasing current over another 30 s. In the sham group, patients received increasing and immediately decreasing current at the beginning and similar application at the end of the stimulation for the purpose of blinding.

### 2.5. Blinding and randomization

This study was performed in a triple-blinded setting. The patient, the tDCS applying physician and the person performing statistics were blinded to group allocation. Blinding of tDCS was performed using the study mode of the NeuroConn DC Stimulator^®^. A block-randomization was used with blocks of six to ensure comparable group sizes in case of early stop of the study. Randomization was performed using a computer-generated random list for patient allocation with four blocks with size of six provided by study statistician. After inclusion, patients were treated following the randomization list. For allocation concealment, block sizes and study randomization list were prepared blinded to study physicians.

### 2.6. Historic control for comparison non-stimulation and stimulation

To explore the intrinsic effect of the stimulation setting, we compared tDCS patients with patients without tDCS obtained in a historic cohort [NCT03066037, report in (10)] with the same infiltration techniques applied in the same outpatient clinic.

### 2.7. Follow-up

Patients were followed-up at 1, 3, and 6 months after completion of the combined stimulation and infiltration series. Follow-Up was performed *via* telephone calls using the German Pain Questionnaire [daily report form, version 2007 (49)]. Furthermore, patients' records at the outpatient clinic were screened whether and when study patients received a new infiltration series outside the study after completion of stimulation and infiltration series.

### 2.8. Statistics

All statistical analyses were performed using SPSS 29. Descriptive data was summarized using mean and standard deviation or median and range depending on scale level and distribution. Analysis of immediate tDCS effect was performed using data from each session. For analyses of statistical significance, NRS-scores were explored using the exact Wilcoxon-signed-rank-test for paired data. To analyse independent groups of ordinal variables, Mann-Whitney-test or Kruskal-Wallis-test was applied, as appropriate. Distribution of continuous data was examined with graphical exploration and Kolmogorov-Smirnov-test. For binary data, Fishers exact test was applied. To describe odds between groups, Mantel-Haenszel estimation was performed. All statistical significance tests used a two-sided alpha level of < 5% and were intended as exploratory in this pilot trial. Similar to previous studies, we defined responders as patients with a pain reduction measured in NRS of at least 50% (9, 10, 16). Patients treated per protocol were included into analysis (*n* = 13). The study was a priori planned to explore a clinical meaningful difference in pain reduction measured in NRS (0.33 vs. 0.5 pain, SD ±0.1) with a power of 0.8 resulting in a number of 24 patients to be randomized. In 2019, the study was temporarily *on hold* due to explore unexpected high rates of drop outs (4 out of 17), however, restart of this trial was not feasible due to ongoing restriction to perform studies during COVID-19 pandemic and thus terminated.

## 3. Results

**Figure 1:**
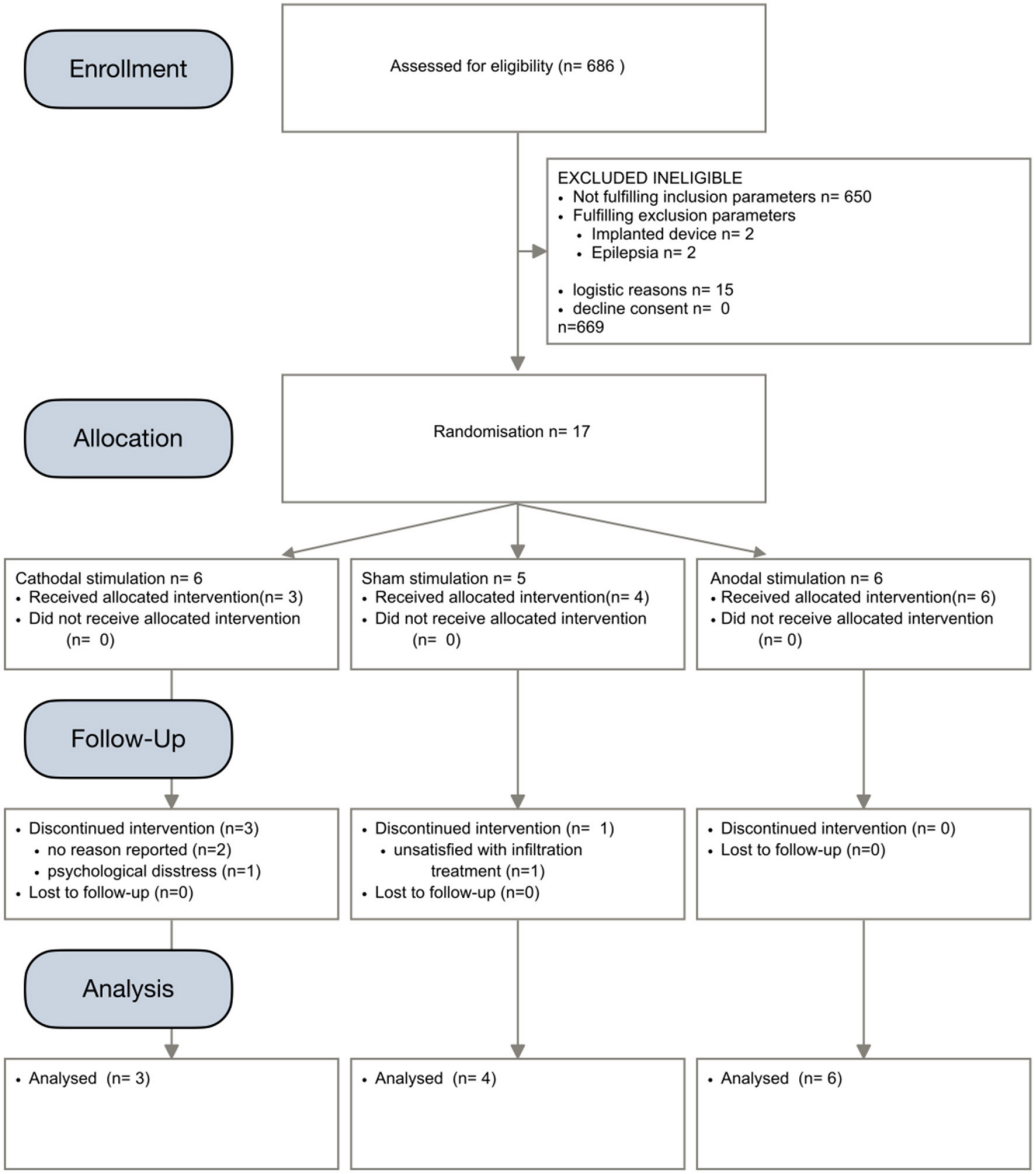
CONSORT chart indicating process of patient recruitment for this study.

Altogether, 686 cases presenting at the pain outpatient center of Charité Virchow Klinikum were pre-screened. Most of these patients did not receive invasive treatment. Patients with refractory cranial pain syndromes scheduled for local infiltration series between June 2016 and March 2019 were screened. We identified 36 patients fulfilling inclusion criteria. After excluding 19 ineligible patients, 17 patients were randomized into the three study groups (cathodal *n* = 6, sham *n* = 5, cathodal *n* = 6). Of these, four patients withdrew their study participation. Thus, thirteen patients were included into analysis (Figure 1). Last follow-up ended in May 2019.

### 3.1. Patients' conditions

Included patients suffered from either trigeminal neuralgia or persistent idiopathic facial pain (PIFP) (basic characteristics, Table 1).

**Table 1:** Basic characteristics for *N* = 13 patients included with refractory neuropathic pain syndromes in the head-neck area.

**Variable**	***N =* 13 patients**
Female gender *n* (%)	7 (53.8%)
Age in years mean (±SD) median (25–75% quartile)	61.08 (±13.77) 58 (IQR 50.0-75)
Duration onset of pain until first infiltration (months) (*N =* 12), median (quartiles)	5 (0.31-19.75)
**Medication at the beginning of infiltration series** ***n*** **in %**	
WHO I: *n* (%)	8 (61.5%)
WHO II: *n* (%)	3 (23.1%)
WHO III: *n* (%)	2 (15.4%)
**Co-analgesic drugs**	
Antidepressants *n* (%)	8 (61.5%)
Antiepileptics *n* (%)	12 (92.3%)
Depressions *n* (%)	2 (15.4%)
**Neuropathic pain classified following ICHD 3, given in** ***n*** **(%)**	
Trigeminal neuralgia, 13.1	10 (76.9%)
Persistent idiopathic facial pain (PIFP), 13.11	3 (23.1%)

### 3.2. Blockade technique

Most patients received a blockade at the sphenopalatine ganglion (SPG) *n* = 11 (84.6%) as main infiltration site. Ganglionic local opioid analgesia (GLOA) infiltration [*n* = 1 (7.7%)] and infiltrations at the N. occ. major [*n* = 1 (7.7%)] were seldom reported as main infiltration site. For SPG blockade local anesthetics (2–3 ml bupivacaine 0.25%) was applied *via* infra-zygomatic injection. For GLOA infiltration, lipophilic opioids and local anesthetics (5 ml 0.5% bupivacaine and 0.03 mg buprenorphine) were injected close to paravertebral cervical ganglions. In patients with mononeuropathic pain patterns, nerve blocks with local anesthetic (ropivacaine 0.2%, 3 ml) were used. Included patients received 2–10 infiltrations and stimulations in a series with median 9 infiltrations and stimulations (IQR 6–10). All 13 per-protocol treated patients received 20 min of tDCS before each infiltration in the series.

### 3.3. Change in pain

**Figure 2:**
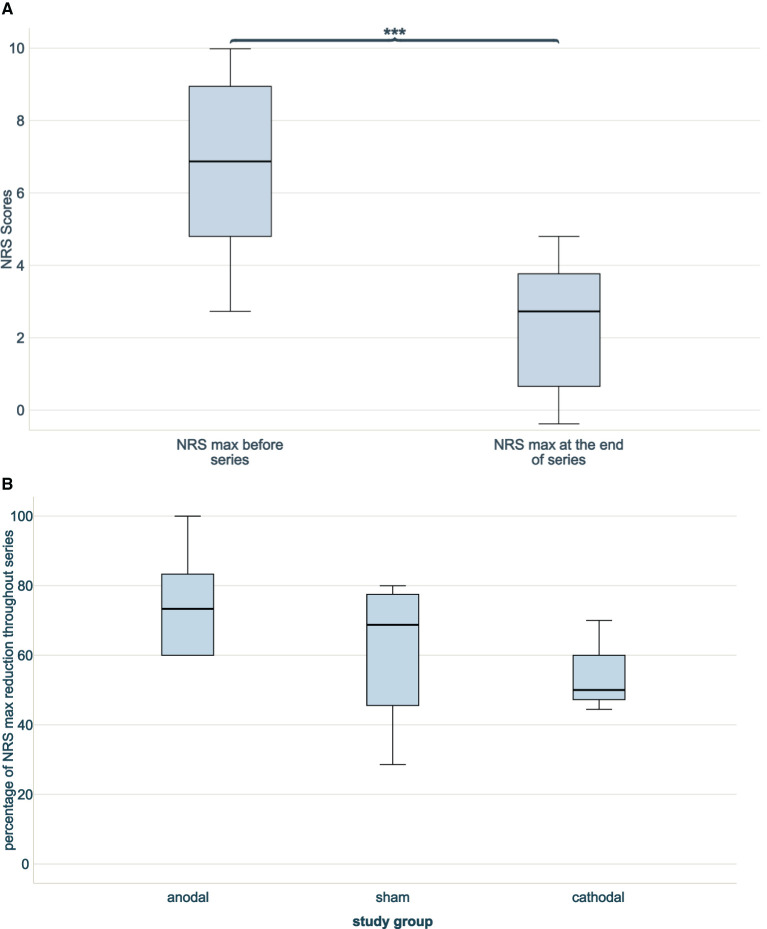
NRS scores through series without dropouts. **(A)** Two boxplots indicating NRS max at beginning and at the end of combined infiltration and stimulation series. A significant NRS reduction was achieved (^***^p < 0.001), *n* = 13. **(B)** Three boxplots showing the percentages of reduction of NRS score at the end of infiltration and stimulation series compared to the beginning between blind groups (anodal *n* = 6, sham *n* = 4, cathodal *n* = 3). No difference in reduction was noted (*n* = 13, *p* = 0.532).

The NRS score before infiltration and stimulation series for per-protocol patients were at median 7 (IQR5.00–9.50). There was no significant difference between dropouts and non-dropouts (*p* = 0.249). Throughout series, there was a significant overall decrease of NRS scores in treated patients (before median 7 (IQR 5.00–9.50), at the end of series median 3 (IQR 1.00–4.00), *p* < 0.001; see Figure 2A).

The NRS scores before intervention were comparable between stimulation groups (*p* = 0.181). NRS scores decreased in all three study groups throughout series. The anodal stimulation group started with lowest NRS scores before series 5.50 (median, IQR 4.50–7.00) and achieved lowest NRS scores after series 1.00 (median, IQR 0.75–4.00), resulting in the highest relative NRS reduction throughout series of 73.33% (median, IQR 50.00-87.50%). The cathodal stimulation group had higher NRS scores before series 9.00 [median, (IQR 8.00–9.00)] and after series 4.00 (median, IQR 3.00–4.00) and, thus, lowest relative NRS reduction 50.00% (median, IQR 44.44–50.00%). The NRS scores in the sham group before series were 7.50 (median, IQR 4.75–9.50), after series 2.50 (median, IQR 1.25–4.50) and a consequent relative NRS reduction of 68.75% (median, IQR 37.05–78.75%). The relative NRS reduction throughout series did not differ significantly between the study groups (*p* = 0.532; see Figure 2B).

### 3.4. Analysis of response

76.9% of patients had a 50% NRS reduction throughout series. Response of 50% NRS reduction was highest in anodal group (83.3%), followed by sham group (75.0%) and cathodal group (66.7%). Differences in response rate were non-significant (*p* = 0.850). Odds ratio in the anodal group for 50% NRS reduction was 1.667 (CI 0.074–37.728) and in the cathodal group 0.667 (CI 0.025–18.059) compared with sham group.

The time to the event of 50% NRS reduction was at Median 3 sessions (IQR 2–3). The time to event was shortest in the cathodal group (median 2, IQR 2–2), followed by sham (median 3, IQR 2.25–3) and anodal group (median 3, IQR 2.75–3.50). Differences in time to event were not significant between stimulation groups (*p* = 0.644).

With a response equalling 50% NRS reduction achieved in 75% of patients in the sham group and an odds ratio of 1.667 in the anodal group, the number needed to treat with anodal stimulation as verum treatment equals NNT = 12.

### 3.5. Course of pain scores throughout series

**Figure 3:**
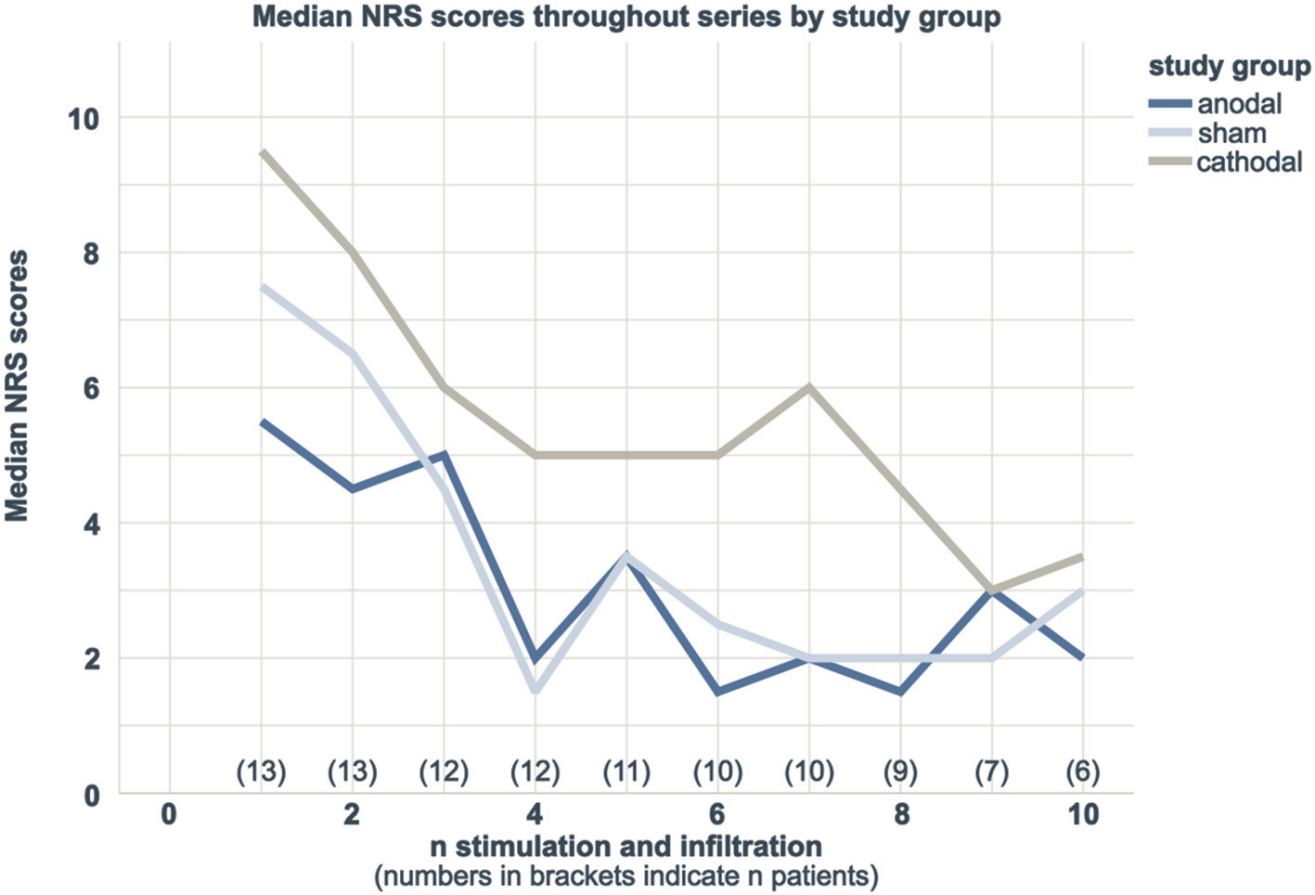
Course of median NRS max values throughout series by study group.

All three subgroups reported an overall NRS reduction throughout series. Course of reported median NRS scores are indicated in Figure 3.

### 3.6. Subgroup analysis of neuropathic pain diagnosis

Overall, 10 patients suffered from trigeminal neuralgia whereas 3 patients had a diagnosis of PIFP. Differences in relative reduction of pain intensity over time were not significant (*p* = 0.469, see Supplementary Figure 2 and Supplementary Table 1).

### 3.7. Effect of sole tDCS stimulation

Overall, there was a significant immediate effect of tDCS before performance of local anesthesia on pain intensity: NRS scores were reduced in median 1 point (IQR 0–2; *p* < 0.001). Nevertheless, no difference between anodal, cathodal or sham stimulation was noted (*p* = 0.482).

### 3.8. *Post-hoc* sample size calculation

Based on the relative NRS reduction throughout series comparing anodal treatment and sham, a sample size calculation for a confirmatory study was performed. A sample size of 93 patients is necessary to achieve level of significance in a confirmatory study. Using a conservative estimation including the drop-out rate of this study with 23.5%, altogether 120 patients would necessary to be randomized.

### 3.9. Analysis of side-effects

Of included per-protocol treated patients, 76.9% (*n* = 10) reported some kind of side-effects due to tDCS. A prickling (53.8%, *n* = 7) or burning sensation (53.8%, *n* = 7) was the most common side-effect. Mild local pain (15.4%, *n* = 2) and skin redness (7.7%, *n* = 1) was reported seldom. No patient reported increased headache due to stimulation. There was no severe side-effect or reported drop-out due to side-effects.

### 3.10. Drop-outs

Four patients dropped out of the study. One reported psycho-social distress not related to the study as the reason for dropout. One patient reported non-sufficient effect of the infiltration and two did not report a reason. Notably, most patients dropped out of the cathodal (*n* = 3) group followed by the sham group (*n* = 1). No patient dropped out of the anodal group.

### 3.11. Comparison non-stimulation and stimulation

**Figure 4:**
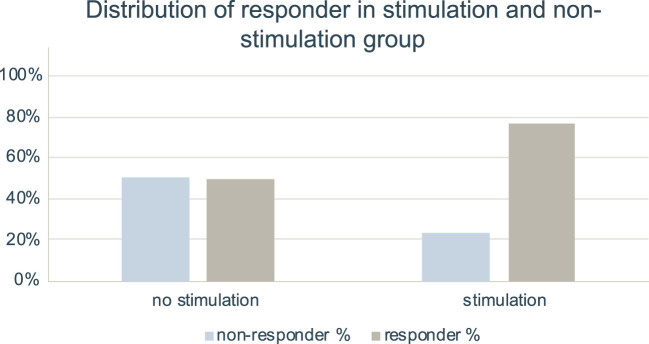
Distribution of responder in stimulation and non-stimulation setting. Bar charts indicating percentage of responder and non-responder with 50% NRS reduction in group with no stimulation setting (*n* = 83) and in group with stimulation setting (*n* = 13). Data for infiltration series without stimulation setting was taken from our previous study (10).

The differences in relative NRS reduction between non-stimulation group (*n* = 83, Median 44.44%, IQR 0.00–70.00%) and stimulation group (*n* = 13, Median 66.66%, IQR 47.22–80.00%) were not significant (*p* = 0.054). A higher proportion of responders with 50% NRS reduction was noted in group with stimulation setting (*n* = 10, 76.9%) than in non-stimulation group (*n* = 41, 49.4%, *p* = 0.079; see Figure 4). This results in an OR of 3.41 for stimulation vs. non-stimulation setting and a subsequent NNT by stimulation setting of 3.63.

### 3.12. Follow-up analysis

There was no statistically significant difference in tDCS study groups regarding maximum pain measured in NRS at 1 month follow-up time point (*p* = 0.645), three-month follow-up time point (*p* = 0.626) and 6 month follow-up time point (*p* = 0.835). There was as well no statistically significant difference in tDCS study groups regarding average pain measured in NRS at 1 month follow-up time point (*p* = 0.404), 3 month follow-up time point (*p* = 0.618) and 6 month follow-up time point (*p* = 0.632).

At 1 month follow-up, there was a significant difference in mental distress between subgroups with cathodal stimulation group having the highest rating in impairment [anodal median 3 (IQR 0–8); sham median 0 (IQR 0–0); cathodal median 8 (IQR 5–8); *p* < 0.05]. In both, impairment in daily activities [anodal 0 (IQR 0–5); sham median 1 (IQR 0.25–1.75), cathodal median 5 (IQR 2–5); *p* = 0.231] and endurance of pain [anodal median 2 (IQR 1.5–2.5); sham median 2 (IQR 1.25–2), cathodal median 3 (IQR 2–3); *p* = 0.154] cathodal stimulation apparently showed worse scores. At both, 3 and 6 months follow-up, we observed no statistical differences in impairment in daily activities, mental distress and endurance of pain.

In total, three patients received additional local infiltration series outside the study after completion of stimulation and infiltration series. The patients with additional series were equally distributed among the study groups (anodal *n* = 1, sham *n* = 1 and cathodal *n* = 1) and the time to the series (median 12, IQR 11–12) did not differ between subgroups (*p* = 0.368).

### 3.13. Analysis of self-assessment

To determine patient blinding, patients were asked in a self-assessment to which group they belong. Group assignment and self-assessment to active or sham stimulation did not show any statistical association (*p* = 0.429).

## 4. Discussion

As the main findings of this randomized controlled pilot trial, we observed tDCS to be safe and feasible applied in multimodal pain management and embedded in infiltration series for neuropathic cranial pain. Nevertheless, effect size to potentially achieve a 50% reduction in pain intensity beyond placebo effect was low with an odds ratio of 1.667 and an estimated number needed to treat of 12. Furthermore, we were able to quantify probable intrinsic placebo effects of tDCS setting with an OR of 3.41 and to show effective blinding measures for further trials in this area.

Adjunctive treatment with tDCS in pain disorders is an emerging field in pain research (18). The combination of both infiltration series and tDCS was performed in this pilot study. This approach seemed promising, since changes in resting state functional connectivity in areas relevant for pain was noted after tDCS and local anesthesia infiltration (13, 25, 39). Although there was an overall significant NRS reduction throughout series in this trial, differences of this reduction between study groups did not reach statistical level of significance. Nevertheless, highest proportion of NRS reduction was noted in the anodal tDCS group and thus, might support the use of anodal M1-stimulation 2 mA for 20 min. This finding goes along with other tDCS studies in the field, stating that this stimulation setting seems to be standard treatment in pain studies evaluating tDCS effects (18). Most studies included in a Cochrane-Review used 2 mA stimulation over 20 min. In that review, single tDCS resulted in a reduction in pain intensity of 0.82 (95% CI 0.42–1.2) points, or a percentage change of 17% (95% CI 9% to 25%) of the control group outcome (19).

From our results, an NNT of 12 can be estimated when comparing effects of anodal tDCS vs. sham-tDCS in our specific chronic pain patient population. A randomized clinical trial with 59 participants with tDCS as add-on treatment for bipolar depression showed an NNT of 5.8 in primary outcome defined as a change from baseline 17-item Hamilton Depression Rating Scale (56). This may indicate that effects of tDCS on pain might be lower than effects in other fields. The assumed effect of tDCS in our study in patients with refractory neuropathic cranial pain syndromes goes in line with the results of other studies. Treatment with patient-conducted anodal tDCS has shown beneficial effects in patients with trigeminal neuralgia: pain intensity was significantly reduced after 2 weeks of treatment (anodal 6.7 ± 1.3 (pre) to 5.5 ± 2.3 (post); sham 7.2 ± 1.2 (pre) to 7.8 ± 1.8 (post), *p* = 0.008) (57). This study by Fitzgibbon et al. highlights another benefit of tDCS: self-applying tDCS by patients themselves and, thus, enhancing self-efficacy (18).

Effects of tDCS on pain could be partly attributed to changes in endogenous inhibitory pathways (28). In a positron-emission tomography study, Garcia-Larrea et al. showed that most changes in cerebral blood flow attributed to electrical stimulation of the precentral gyrus was noted in the ventral-lateral thalamus hypothesizing that this may reflect cortico-thalamic connections (58). Thalamo-cortical connections seem to play a crucial role in pain (59–61). A different approach explains effects by modulation of cortical plasticity (18). A resting state functional MRI study with fibromyalgia patients showed changes in cortical plasticity correlated with pain reduction in both, sham and active tDCS treatment. Hence, it was hypothesized that there might be a placebo response common to both sham and real tDCS (62). This goes together with results of a PET-MRI trial indicating that acute changes in endogenous μ-opioid receptor mediated neurotransmission are produced by sham-tDCS but enhanced at molecular and clinical levels by real tDCS (63). Although reduction of NRS scores throughout tDCS series in our study should be partly attributed to nerve block techniques (10), we could speculate that μ-opioidergic effects being subclinic in single session tDCS (64) might multiply in subsequent tDCS-series. This repetitive stimulation may be necessary to reverse changes in neuroplasticity especially related to chronic pain. The repetition of stimulation is supported by results from animal data of a chronic neuropathic pain model in rats in which repetitive anodal tDCS over the M1 areal had a longer analgesic effect than single stimulus (65). A systematic review on non-invasive brain stimulation in oro-facial pain stated that higher number of sessions seems to be accompanied by more durable effects (24).

A reduction of 50% pain intensity is common as a description for successful treatment and used in this study (9, 10, 16, 66). When compared to previous data of infiltration series without tDCS, responder rate in this tDCS trial was higher, though statistically non-significant (10). This suggests a strong placebo effect, which was calculated with an NNT of 3.6. Therefore, the overlying placebo effect seems to be stronger than the inherent effects provided by tDCS. Furthermore, we found an immediate effect of tDCS on pain intensity which did not differ between cathodal, anodal and sham stimulation. This finding supports the idea of a direct placebo effect mediated by the tDCS setting.

It has been reported that invasive procedures might have more powerful placebo effects than less invasive procedures. Expectancy is one of the most powerful causes of placebo effects. Infiltration and stimulation with tDCS enhances expectations regarding pain relief (67). Furthermore, longer studies with more than six weeks of follow-up seem to have a more profound placebo effect (68). Consequently, study designs need to acknowledge strong placebo effects. Interestingly, worse scoring for cathodal tDCS at 1 month follow-up time-point together with relatively high drop-out rate in this group could be suggestive for a deteriorating influence of cathodal M1 stimulation on pain patients. This could be due to inverse effects of excitability of the underlying cortex of anodal and cathodal tDCS stimulation (22, 29). This inverse effect might also explain the higher rated distress in cathodal group since anodal tDCS has been repeatedly reported to have a beneficial effect on depression (69).

### 4.1. Limitations

Although providing insights into tDCS for chronic neuropathic pain patients, this pilot study has some limitations. Especially the small sample size in pilot studies limit generalizability of results. Furthermore, individual pain perception differs between patients and other outcome measurements like change in medication would be an interesting variable to include in future protocols (70). In addition to that, the inclusion and exclusion criteria did not cover all possible confounders (e.g., the effects of pain medication). To cover such unmeasured confounders, the study was performed in a randomized-controlled design with included long term data on patients. Another limitation were differences in the baseline NRS among the different study groups. Though statistically not significant, we used relative instead of absolute NRS reduction and a definition of 50% NRS reduction as a clinically relevant response to address these differences. Regarding suitable outcome measurements, resting pain, maximum pain in exertion as well as frequency of pain attacks and change in pain medication should be assessed.

As blinding is a critical issue in tDCS, we observed sufficient blinding provided by the study mode of the NeuroConn DC-Stimulator^®^. There was no connection noted between the self-assessment of patients and the actual study group. Thus, our results support the use of an increasing and decreasing current at the beginning and end of a sham stimulation to imitate effects of tDCS. This goes with the results of the study by Gandiga et al. who described this sham procedure. In their pooled data analysis including several studies over 3 years with 170 stimulation sessions, there was no difference in the incidence of side-effects between tDCS and sham groups (71). This was contrary to results from a study evaluating differences between sham and active tDCS with 131 patients receiving either type of stimulation; a statistically higher rate of sensory side effects was noted in the active tDCS group (72). Based on our finding, blinding in our tDCS population was sufficient.

### 4.2. Future directions

Future studies could try to lower the barriers to tDCS application with supervised stimulation at home (18). A different approaches is to combine non-invasive brain stimulation with other non-invasive approaches such as neurofeedback to further enhance possible beneficial effects (73). Not only feedback to the patient but also to the tDCS applying in real-time tool could be a new research direction. Thus ongoing brain activity could provide the base for closed-loop technology allowing the delivery of tDCS specific to an individual's internal state (18). Furthermore, future studies should address the limitations reported in our study including small sample size and unmeasured confounders. A sample size calculation for such future studies, revealed the necessity of 120 patients with refractory neuropathic cranial pain to confirm our findings. This is challenging, since infiltration series are only used in patients refractory to standard treatment and thus performed relatively rarely. In 4 years, a study in an university affiliated pain outpatient clinic reported only 74 patients receiving a ganglionic opioid analgesia (GLOA) at the superior cervical ganglion as an infiltration series (16). These numbers are supported by our previous study performed as well in a university affiliated pain outpatient clinic with 83 patients receiving infiltration series in six-and-a-half years (10). Hence, a confirmatory study could only be performed in multicentre study design.

## Data availability statement

Data can be accessed upon request by contacting the last author ST via e-mail: sascha.tafelski@charite.de.

## Ethics statement

This study involving human participants was reviewed and approved by Ethikkommission der Charité – Universitätsmedizin Berlin Campus Charité Mitte, Germany (EA1/031/16). The patients provided their written informed consent to participate in this study.

## Author contributions

JDW: acquisition of data, analysis and interpretation of data, creating of all figures and tables, drafting the article, revising the article critically for important intellectual content, and final approval of the version to be published. JK: acquisition of data, revising the article critically for important intellectual content, and final approval of the version to be published. TF: conception of study, revising the article for important intellectual content, and final approval of the version to be published. CD: revising the article for important intellectual content and final approval of the version to be published. MS: giving advice to conception and design of study, revising the article critically for important intellectual content, and final approval of the version to be published. ST: conception and design of study, analysis and interpretation of data, revising the article critically for important intellectual content, and final approval of the version to be published. All authors contributed to the article and approved the submitted version.
