# Supplementary figures and images for: Supplementing transcranial direct current stimulation to local infiltration series for refractory neuropathic craniocephalic pain: A randomized controlled pilot trial
Source: Front Neurol. 2023 Mar 1;14:1069434. doi: 10.3389/fneur.2023.1069434 (PMC10014889; doi:10.3389/fneur.2023.1069434)

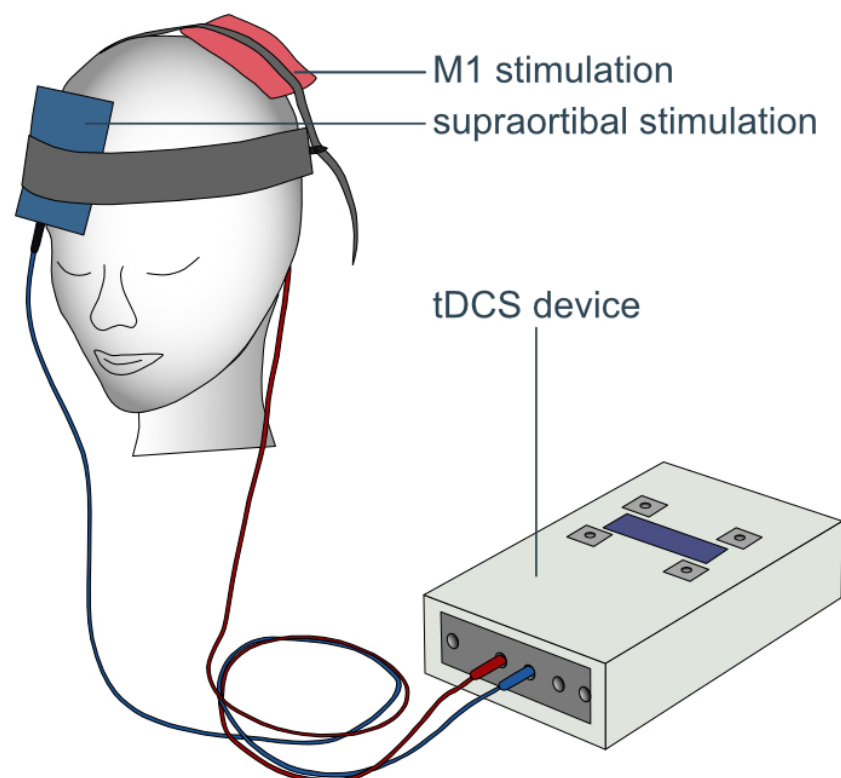

**Suppl. figure 1:** Simplified graphic of the tDCS setup used in this study.

Supplement: Supplementary file 1 [file Image_1.pdf]
