# Supplementary material for: Supplementing transcranial direct current stimulation to local infiltration series for refractory neuropathic craniocephalic pain: A randomized controlled pilot trial
Source: Front Neurol. 2023 Mar 1;14:1069434. doi: 10.3389/fneur.2023.1069434 (PMC10014889; doi:10.3389/fneur.2023.1069434)
Supplement: Supplementary file 2 [file Image_2.pdf]

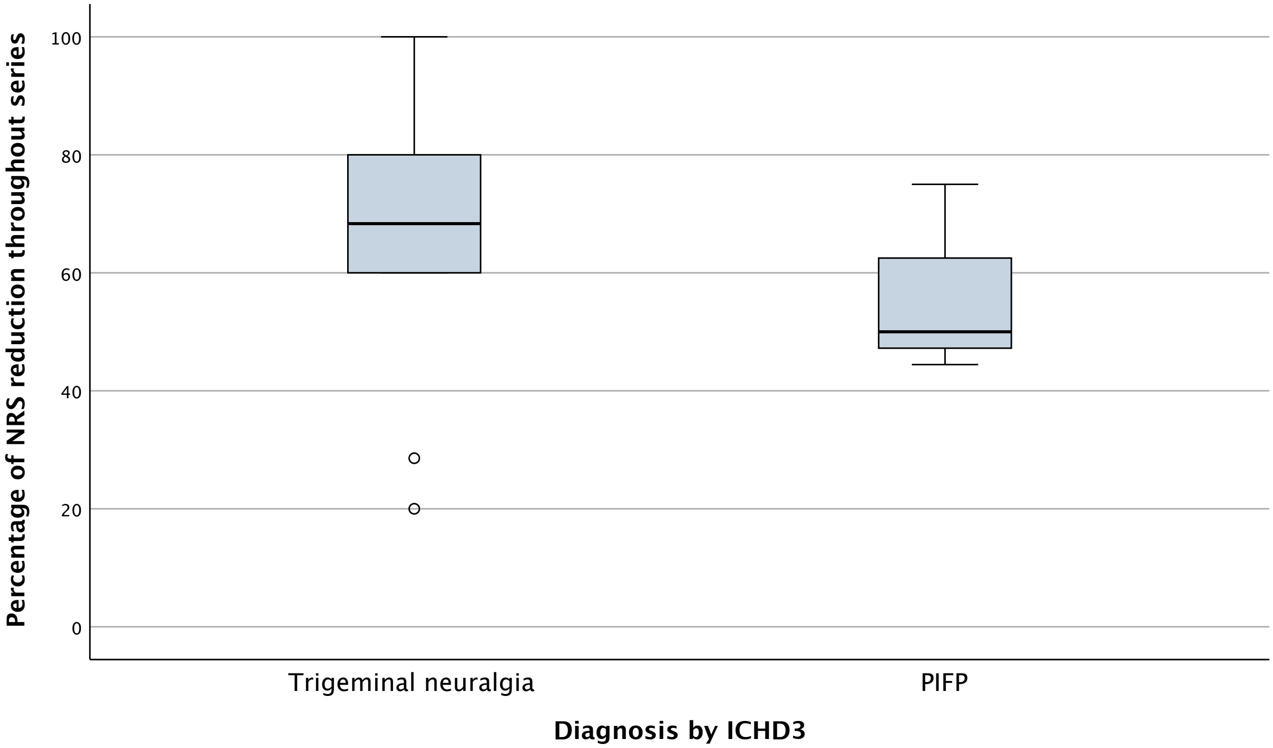

Suppl. figure 2: Percentage of relative reduction of maximum pain measured in NRS throughout series by neuropathic pain diagnosis. Overall, 10 patients suffered from trigeminal neuralgia whereas 3 patients had a diagnosis of PIFP. Differences in reduction were not significant (p=0.469).
