# Supplementary material for: Supplementing transcranial direct current stimulation to local infiltration series for refractory neuropathic craniocephalic pain: A randomized controlled pilot trial
Source: Front Neurol. 2023 Mar 1;14:1069434. doi: 10.3389/fneur.2023.1069434 (PMC10014889; doi:10.3389/fneur.2023.1069434)
Supplement: Supplementary file 3 [file Table_1.pdf]

Suppl. table 1: Percentage of relative reduction of maximum pain measured in NRS throughout series by neuropathic pain diagnosis and stimulation group.

**Median (25-75 quartile)**<br>**n patients in each subgroup**	**Trigeminal neuralgia**	**PIFP**
**Anodal stimulation**	73.3 (60.0-83.3)<br>n=6	-<br>n=0
**Sham stimulation**	62.5 (45.5-71.2)<br>n=3	75.0<br>n=1
**Cathodal stimulation**	70.0<br>n=1	47.2 (44.4-50.0)<br>n=2
